# The Usefulness of the Lateral Femoral Epicondyle as a Landmark for Evaluating Leg Length Discrepancy in Robot-Assisted Total Hip Arthroplasty

**DOI:** 10.3390/jcm14092905

**Published:** 2025-04-23

**Authors:** Dongnyoung Lee, Changhyun Nam, Ji-Hoon Baek, Suchan Lee, Suengryol Ryu, Taehyeon Kim, Jihyo Hwang

**Affiliations:** 1 Joint & Arthritis Research, Himchan Hospital, Seoul 07999, Republic of Korea; fretless@hanmail.net (D.L.); changcape@naver.com (C.N.); jihoon011@naver.com (J.-H.B.); himchanhospital@naver.com (S.L.); oszzang102@naver.com (S.R.); ysunispica@naver.com (T.K.); 2 Department of Orthopaedic Surgery, Gangnam Sacred Heart Hospital, Hallym University College of Medicine, 1 Singil-ro, Yeongdeungpo-gu, Seoul 07441, Republic of Korea

**Keywords:** limb length discrepancy, Mako, lateral femoral epicondyle, total hip arthroplasty

## Abstract

**Objective**: One of the biggest advantages of robot-assisted hip replacement surgery is the reduction in dislocation rates because of proper implant positioning and accurate measurement of the leg length. Therefore, we aimed to investigate the usefulness of using the lateral epicondyle as a landmark to minimize errors in leg length measurement. **Methods**: This retrospective study was conducted between September 2023 and March 2025 and included 24 patients who underwent robotic-assisted total hip arthroplasty (rTHA, Group I). These procedures were performed by two experienced surgeons. The data were compared with results from two additional groups: patients who underwent rTHA using the intrapatella landmark (Group II) and those who underwent conventional total hip arthroplasty (cTHA) without robotic assistance (Group III). Leg length measurements were evaluated using postoperative X-rays and intraoperative robotic monitoring. ANOVA and Student’s *t*-test were used to analyze the significance of the variables (*p* < 0.05). **Results**: The mean X-ray LLD (xLLD) was 1.39 mm (−7.43–11.63 mm) and Mako LLD (mLLD) was 4.77 mm (−6–12 mm) in Group I. The mean xLLD was 3.54 mm (−5.02–13.6 mm) and mLLD was 4.20 mm (−22–14 mm) in Group II. The mean xLLD was 4.06 mm (−8.62–21.2 mm) in Group III. There was no statistical significance between the three groups (*p* = 0.241). **Conclusions**: Using the lateral femoral epicondyle as a landmark for the limb length measurement is a viable alternative to the intrapatella landmark in rTHA. This method may save time and offer and more convenient technique in measuring leg length changes during robotic-assisted total hip arthroplasty.

## 1. Background

Total hip arthroplasty (THA) has been one of the most successful surgical procedures in the field of hip replacement surgery over the past 50 years [1]. However, despite the high success rate of THA, postoperative complications such as leg length discrepancy (LLD), hip dislocation, and nerve injury can negatively impact patient satisfaction after surgery [2,3]. In particular, LLD can lead to decreased hip stability, increased back pain, gait disorders, and litigation [4,5,6,7,8]. Robot-assisted THA (rTHA) enables highly accurate intraoperative LLD measurements, often producing results that are imperceptible to the patient.

However, the conventional Mako surgical reference guide [9] recommends attaching an EKG lead to the intrapatella landmark for LLD measurement. This method presents challenges, as the lead can shift during hip joint reduction when leg traction is applied, and changes in the knee range of motion (ROM) can alter the length of the patellar tendon, affecting the position of the patella. These factors can introduce challenges in accurately assessing leg length changes at the millimeter level.

This study aims to evaluate whether changing the EKG lead attachment site for leg length measurement in Mako rTHA—from the intrapatella landmark to the lateral epicondyle of the distal femur—can more accurately reflect changes in leg length before and after prosthesis placement.

## 2. Materials and Methods

This study retrospectively investigated a total of 26 patients who underwent Mako rTHA (Stryker Orthopaedics, Mahwah, NJ, USA, software version number 4.0) using the intrapatellar landmark at two hospitals between September 2023 and March 2025. The study was conducted in accordance with the Declaration of Helsinki and approved by the Institutional Review Board (116655-01-202310-01).

The procedures were performed by two orthopedic surgeons (Jihyo H and Dongnyoung L), each with over 10 years of experience in THA. All patients underwent history-taking and physical examinations, along with radiographic studies, including preoperative plain anteroposterior (AP) pelvis X-rays, axial X-ray of both hips, preoperative and postoperative hip CT scans, and serial plain hip X-rays.

Patients of all ages and genders were included if they were scheduled for unilateral THA, had grade II or III hip osteoarthritis based on the Tönnis radiographic classification, or had avascular necrosis of the femoral head at Ficat stages IIb and higher, with functional limitations requiring pain medication for at least 3 months. Additionally, only those who could understand information about the use of the new rTHA and provided informed consent were included.

The exclusion criteria for patients were as follows: (1) active infection, (2) significant hip deformity on the contralateral side that could hinder leg length evaluation, (3) any neurological conditions (e.g., cognitive impairment and paralysis) or other diseases (e.g., heart failure and severe lung disease) that could limit postoperative exercise and daily active activity, (4) severe spinal deformities, and (5) history of alcoholism or drug abuse. To compare the usefulness of this new technique (Group I), two additional groups of 26 patients each were included: one group undergoing rTHA using the intrapatella landmark (Group II) and another undergoing conventional THA without robotic assistance (Group III).

The mean age of the patients (Group I) was 58.0 (36–77) years, and the overall mean BMI (Body Mass Index: kg/m^2^) was 24.94 (16.67–31.67). Preoperative diagnoses included osteonecrosis (20 cases, 76.9%), primary osteoarthritis (4 cases, 15.4%), secondary osteoarthritis (1 case, 3.8%), and ankylosing spondylitis (1 case, 3.8%). The mean preoperative LLD was −5.25 mm (−21.72–6.81 mm), with three patients exhibiting a longer leg measurement (2.66–6.81 mm). In all other patients, leg shortening was the cause of LLD.

Radiographic evaluation of LLD was conducted using the picture archiving and communication system (PACS), which has same version of program between two different hospitals. Leg length was measured on an anteroposterior view of the hip joints taken with both lower legs internally rotated by 15°. The measurement was based on the difference between the inferior margin of the ischial tuberosity in the pelvic bone and the lowest point of the lesser trochanter [9]. To reduce inter-observer reliability bias, one surgeon (Jihyo H) measured whole images. The accuracy of mLLD obtained through the new EKG lead attachment site was verified by comparing it with postoperative LLD (xLLD), measured from bilateral pelvic radiographs, with intraoperative LLD (mLLD), displayed on the Mako monitor (calculated by inputting the positions of the check point inserted into the femoral greater trochanter and the EKG lead attached to the lateral femoral epicondyle) after inserting the implant. These results were compared with LLD outcomes in an equal number of patients who underwent THA using the intrapatella landmark and those who underwent conventional THA. Moreover, as anatomical landmarks may be less distinct in obese patients, potentially introducing bias, a comparison was conducted with patients having a BMI of 25 or higher. Radiographic evaluations were performed by the operating surgeons.

## 3. Surgical Procedure

**Figure 1:**
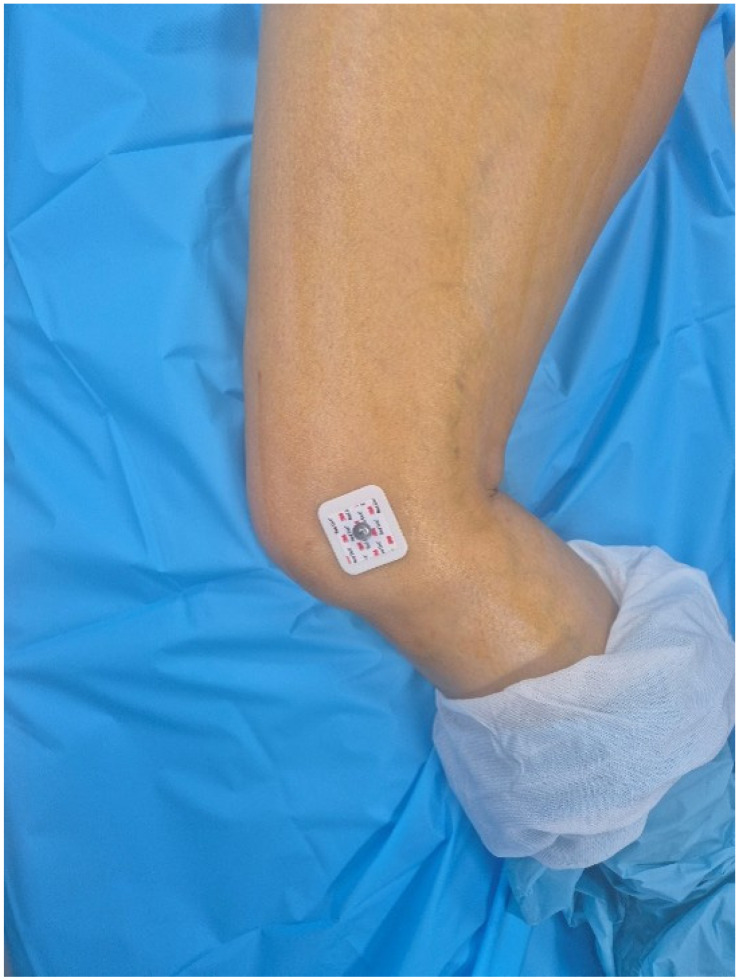
EKG lead attached to the femoral epicondyle.

For robotic-assisted surgery, a monitoring camera for the surgical site was positioned between the 11 o’clock and 1 o’clock positions while the robotic arm was placed in front of the patient. The patient was positioned in the lateral decubitus position for a posterolateral approach. To measure preoperative leg length, instead of attaching the EKG lead to the inferior pole of the patella (as recommended in the Mako surgical reference guide), it was attached to the lateral epicondyle of the distal femur to input the position of the distal femur into the robotic system. The lateral epicondyle of the distal femur was easily palpable due to its prominence, making it straightforward to determine the correct placement, and the lead was secured (Figure 1). The operated area was then rewrapped with surgical tape (Ioban^TM^), followed by sterilization and draping procedures. For a standard Mako rTHA, a stab incision was made on the lateral aspect of the iliac crest, followed by the insertion of a bone pin and the placement of the pelvic array. After incising the surgical site, the modified Gibson approach was used to access the hip joint. Before dislocating the hip to measure preoperative leg length, a checkpoint was inserted into the femoral greater trochanter (GT). The distances from the pelvic array to the GT and from the pelvic array to the EKG lead attached to the distal femur were then entered into the Mako robotic system to establish baseline LLD measurements before implant placement. While assessing the tension in the soft tissues around the proximal femur, the surgeon gradually released soft tissues surrounding the femoral GT and neck (joint capsule, piriformis, gemellus, and obturator internus muscles) to dislocate the hip. Once the femoral head was exposed, the femoral neck was osteotomized, and the femoral head was removed. After inserting an additional pelvic check point in the superolateral portion of the acetabulum, registration and verification were performed for 15 intraarticular points and 15 extraarticular points. Then, reaming was conducted using an acetabular reamer attached to the robot arm, and the real cup was inserted with the planned inclination and anteversion. The femoral stem insertion site was prepared manually, and the femoral stem and head were selected and implanted to achieve hip reduction. Postoperatively, the distance from the femur check point to the EKG lead on the distal femur was measured to confirm the postoperative LLD results displayed on the Mako monitor. Across all patient groups, the Trident PSL HA™ (Stryker, Warsaw, IN, USA) acetabular cup, modular dual mobility (Stryker, Warsaw, IN, USA) liner, and Accolade II™ (Stryker, Warsaw, IN, USA) femoral implant were used. All surgeries followed an acetabulum-first approach and were performed using the express workflow mode.

## 4. Statistical Analysis

Continuous variables were analyzed using the independent *t*-test, while comparisons among the three groups were performed using the analysis of variance (ANOVA). A *p*-value of <0.05 was considered statistically significant. All statistical analyses were conducted using Statistical Package for the Social Sciences version 27 (SPSS v27, Chicago, IL, USA).

## 5. Results

No patients in the study experienced serious complications that could have influenced the results. The mean age of patients in Group I was 58.0 years (range, 36–77), with 13 males (50%) and 13 females (50%). The patients had a mean height of 162.8 cm (149–175 cm), a mean weight of 66.3 kg (48.7–97 kg), and a mean BMI of 24.9 Kg/m^2^ (16.7–31.7 Kg/m^2^). The mean operative time was 68.0 min (50–100 min), and the mean estimated blood loss was 244 mL (20–700 mL) (Table 1).

**Figure 2:**
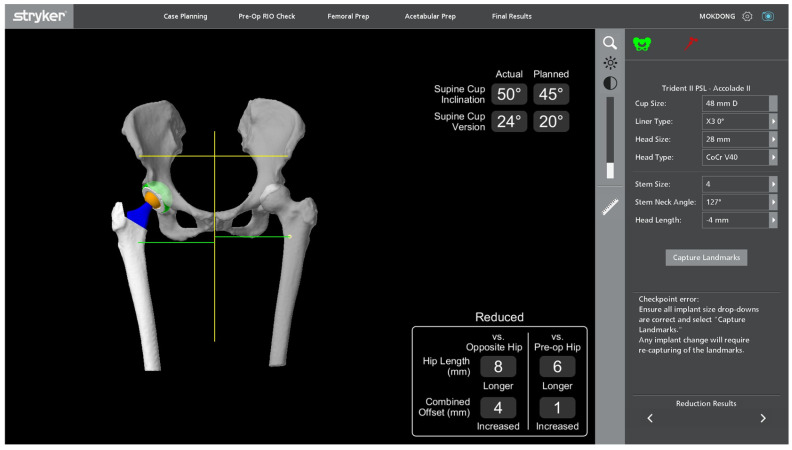
LLD (mLLD) checked using the Mako system displayed on the monitor in the operating room after THA implantation.

**Figure 3:**
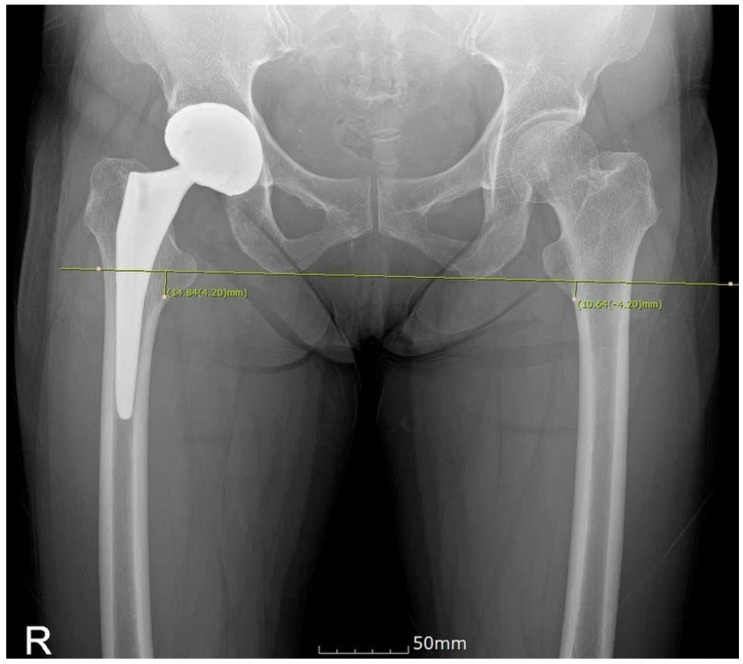
Postoperative LLD (xLLD) measured using PACS.

After changing the EKG lead attachment site in Mako-assisted THA from the intrapatella landmark to the lateral femoral epicondyle, we compared intraoperative LLD measurements (mLLD) (Figure 2) with postoperative X-ray-based LLD measurements (xLLD) using PACS (Figure 3). The mean mLLD was 4.8 mm (−6–12 mm), while the mean xLLD was 1.43 mm (−7.43–11.63 mm), showing that X-ray-based LLD measurements yielded better results than mLLD (Table 2).

Although obesity could potentially reduce the accuracy of mLLD measurements, a comparison between 13 patients with a BMI of ≥25 and 13 patients with normal weight showed no significant difference in mean LLD measurements (Table 3).

For patients who underwent cTHA, postoperative xLLD ranged from −8.62 to 21.2 mm, with a mean of 4.06 mm. No statistical differences in LLD were found among the three groups (Table 4).

## 6. Discussion

rTHA offers several advantages over conventional manual arthroplasty. rTHA enables the measurement of an impingement-free virtual ROM, reducing the risk of dislocation, and ensures precise correction of LLD. LLD following THA can lead to changes in hip biomechanics, dysfunctional gait, lower back pain, sciatica, instability, and early implant loosening [10]. The reported incidence of LLD after THA ranges from 1% to 30%, with discrepancies varying from 3 to 17 mm [11].

In this study, we selected the lateral epicondyle as the distal landmark for LLD measurement instead of the conventional intrapatella landmark, making this the first investigation to compare these two methods. While intraoperative Mako LLD measurements showed no significant differences, postoperative X-ray LLD measurements demonstrated superior accuracy when using the lateral epicondyle landmark compared to the intrapatella landmark, suggesting that the lateral epicondyle serves as a more precise distal landmark for reducing LLD measurement errors.

**Figure 4:**
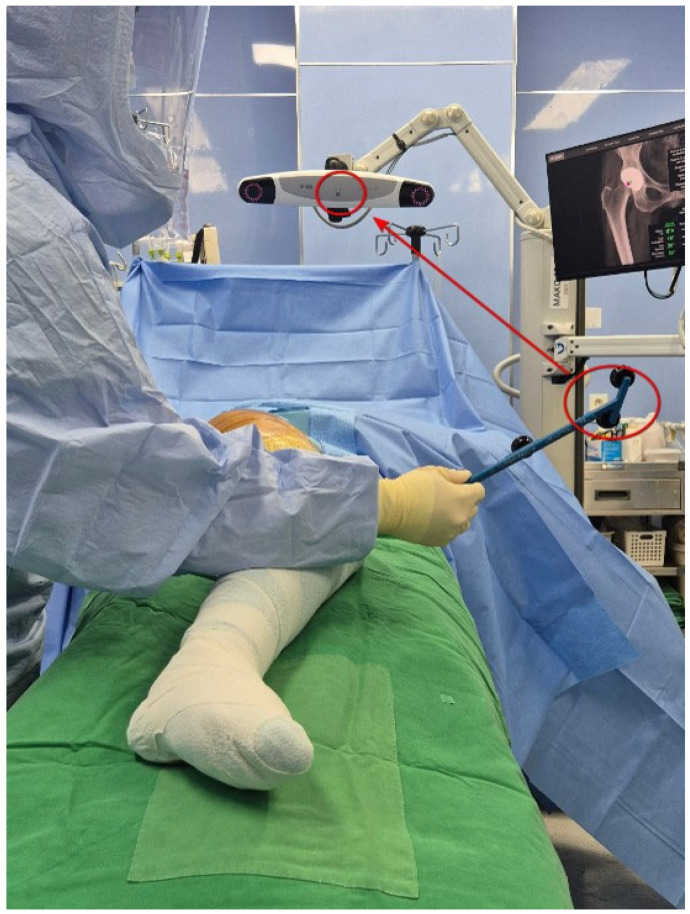
LLD measurement using the Mako probe after attaching the EKG lead to the patellar inferior pole. The circle on the lead held by the assistant indicates the Mako probe. The circle over the patient indicates the EKG lead. The arrow shows that when the Mako probe is placed on the patellar inferior pole, the EKG lead measures the leg length discrepancy (LLD).

**Figure 5:**
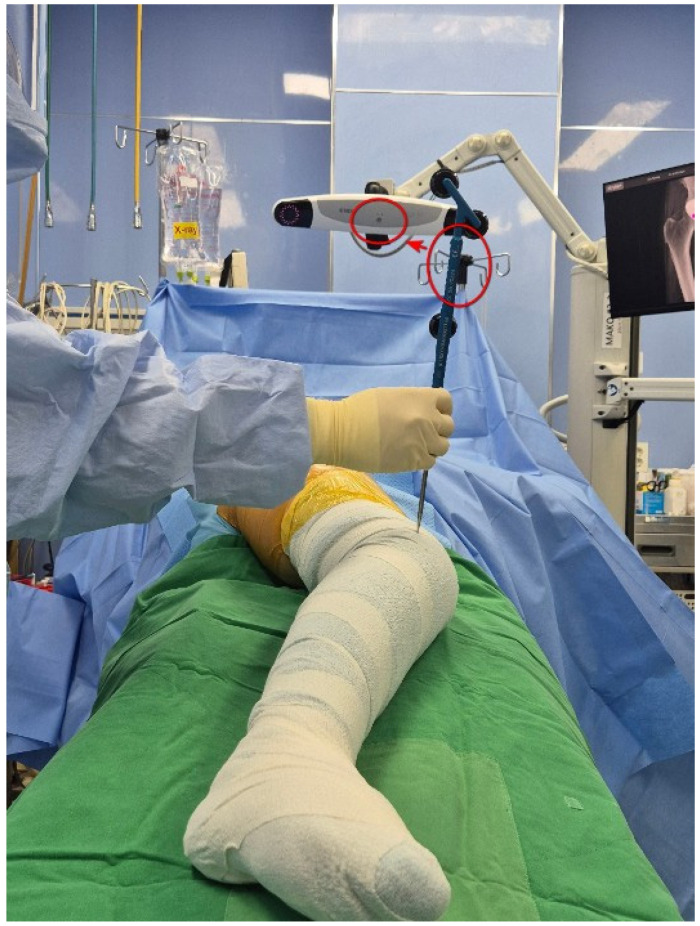
LLD measurement using the Mako probe after attaching the EKG lead to the lateral femoral epicondyle. The circle on the lead held by the assistant indicates the Mako probe. The circle over the patient indicates the EKG lead. The arrow shows that when the Mako probe is placed on the patellar inferior pole, the EKG lead measures the leg length discrepancy (LLD).

In practice, the lateral femoral condyle is used as a landmark in revision total knee arthroplasty to restore the joint line 2.5 cm below the lateral femoral epicondyle after removing the femoral implant. In primary TKA, the lateral femoral epicondyle is known to be a more prominent and easily identifiable structure than the medial epicondyle along the transepicondylar axis, which is used to determine femoral external rotation [11]. In the posterior-lateral approach to THA, the patient is positioned laterally, aligning the lateral femoral epicondyle with the extension of the surgical incision. But compared to the infrapatella landmark, the lateral epicondyle can be vague among obese patients or confused with osteophytes with degenerative osteoarthritic change around the knee. If the patient had a distal femoral osteotomy, the landmark of the lateral epicondyle can also be problematic. It suggests that the lateral epicondyle landmark can be an alternative option, not a routine technique. When the EKG lead is attached to this site (Figure 4), it is easier to orient the Mako probe toward the Mako monitor for position input compared to when the EKG lead is attached below the patella (Figure 5).

Furthermore, during hip joint reduction after trial implant insertion, the leg is pulled downward for traction. If the EKG lead is attached to the patellar inferior pole at this stage, its position may shift during traction. As LLD is assessed at the millimeter level, even slight movements can introduce errors.

Buford et al. [12] reported that knee ROM can cause variations in patellar tendon length, ranging from a minimum of 27 mm to a maximum of 51 mm. If the leg length comparison before and after implant insertion is not checked in the exact same knee position while the EKG lead is attached to the patellar inferior pole, there is a high likelihood of measurement errors due to changes in the length of the patellar tendon. Wylde et al. reported that 30% of patients who underwent THA perceived a leg length difference, but only approximately 1/3 (36%) of these cases actually had a structural LLD on radiologic imaging [13]. In cases without structural LLD, functional compensation typically resolves within 6 months postoperatively [11]. According to O’Brien’s study, patients perceived a shortening of >10 mm or a lengthening of >6 mm after THA. Tsang et al. found that differences of <5 mm were asymptomatic, whereas differences exceeding 2 cm were almost always symptomatic [14,15].

Numerous studies have reported that robotic assistance in THA helps reduce LLD [16,17]. Nakamura et al. [18] found a significant reduction in LLD in rTHA patients among a cohort of 146 THA cases. Honl et al. [19] reported that the rTHA group had a mean LLD of 0.18 ± 0.30 cm, compared to 0.96 ± 0.93 cm in the manual THA group, demonstrating superior outcomes with robotic assistance. Additionally, a comparison between rTHA and fluoroscopic-assisted THA demonstrated similar LLD and outlier rates in both groups [20]. In this study, LLD using the intrapatellar landmark (1.39 mm) was significantly reduced compared to the manual THA group (4.06 mm).

However, as this study was conducted on only 26 patients, the small sample size may limit the statistical power of our conclusions, and this is the biggest limitation; robust data are still being gathered for future studies. Furthermore, there is a potential issue of intra-observer variability, which means the results can be variable by the analyzer. However, compared to the conventional intrapatella landmark, the lateral epicondyle is easier to prove and can be relatively easily detected, even in obese patients, making it a viable alternative technique for measuring hip length using the Mako robot. Additionally, attaching the EKG lead to the lateral femoral epicondyle has various advantages over attachment to the patellar inferior pole: (1) it is an anatomically prominent structure, making it easier to locate; (2) the probe used to register the EKG lead position is more directly aligned with the monitor, facilitating more convenient input; (3) by attaching the EKG lead to an area unaffected by knee ROM, errors due to changes in knee position can be minimized; and (4) it can reduce the possibility of errors caused by changes in the EKG lead position due to hip joint reduction movements after trial component insertion.

## 7. Conclusions

In this study, we attached the EKG lead to the lateral femoral epicondyle for intraoperative LLD evaluation in rTHA. We confirmed that the intraoperatively measured lateral epicondyle technique was comparable to the conventional intrapatella landmark technique. There are many advantages compared to the intrapatella landmark technique. The lateral epicondyle landmark technique can be a good alternative landmark during rTHA.

## Figures and Tables

**Table 1:** Demographic data from Groups I, II, and III.

Patients (26)	Group I	Group II	Group III	*p*-Value
Age (years)	58.0 ± 11.74	58.73 ± 11.48	57.9 ± 12.51	0.968
Sex (male–female)	14:12	14:12	17:9	
BMI (kg/m^2^)	24.94 ± 3.38	24.40 ± 9.46	24.20 ± 1.86	0.762
Operating time (minutes)	68.03 ± 18.59	77 ± 28.28	65.88 ± 3.53	0.008
EBL (mL)	244.23 ± 195.24	150.76 ± 318.19	172.30 ± 0	0.037
Diagnosis				
ONFH	20 (76.9%)	18 (69.2%)	16 (61.5%)	
1ry OA	4 (15.4%)	6 (23.1%)	1 (3.8%)	
2ndary OA	1 (3.8%)	2 (7.7%)	9 (34.6)	
AS	1 (3.8%)			

Data are presented as mean ± SD unless otherwise indicated. BMI: Body Mass Index; EBL: estimated blood loss; OA: osteoarthritis; ONFH: osteonecrosis of femoral head; AS: ankylosing spondylitis; SD: standard deviation.

**Table 2:** Postoperative X-ray LLD and intraoperative Mako LLD in Group I.

	xLLD (mm)	mLLD (mm)	*p*-Value
LLD	1.39 ± 4.62	4.76 ± 3.69	0.006

Data are presented as mean ± SD unless otherwise indicated. LLD: leg length discrepancy; SD: standard deviation, paired *t*-test.

**Table 3:** LLD difference between the obesity (BMI > 25) and normal weight (BMI < 25) in Group I.

	BMI > 25	BMI < 25	*p*-Value
xLLD (mm)	0.17 ± 4.62	2.62 ± 4.49	0.184
mLLD (mm)	4.46 ± 4.03	5.07 ± 3.45	0.680

Data are presented as mean ± SD unless otherwise indicated. BMI: Body Mass Index; SD: standard deviation, paired *t*-test ADDIN.

**Table 4:** Comparison of LLD between three groups.

	xLLD (mm)	mLLD (mm)	*p*-Value
Group I	1.39 ± 4.63	4.76 ± 3.69	0.241
Group II	3.54 ± 4.17	4.20 ± 6.42	
Group III	4.06 ± 7.53		

Data are presented as mean ± SD unless otherwise indicated. SD: standard deviation, ANOVA post hoc Scheffee.

## Data Availability

The data presented in this study are available upon reasonable request from the corresponding author.
